# *Artocarpus lakoocha* Roxb. and *Artocarpus heterophyllus* Lam. Flowers: New Sources of Bioactive Compounds

**DOI:** 10.3390/plants9101329

**Published:** 2020-10-09

**Authors:** Arun Kumar Gupta, Muzamil Ahmad Rather, Avinash Kumar Jha, Abhinay Shashank, Somya Singhal, Maanas Sharma, Urbi Pathak, Dipti Sharma, Andrea Mastinu

**Affiliations:** 1 Department of Food Engineering and Technology, Tezpur University, Assam 784028, India; arunk@tezu.ernet.in (A.K.G.); avinash@tezu.ernet.in (A.K.J.); somyasi@tezu.ernet.in (S.S.); manass@tezu.ernet.in (M.S.); 2 Department of Molecular Biology and Biotechnology, Tezpur University, Assam 784028, India; muzamil@tezu.ernet.in; 3 Department of Dairy Science and Food Technology, Institute of Agricultural Sciences, Banaras Hindu University, Varanasi 221005, India; abhinay.shashank@bhu.ac.in; 4 Department of Food Science, ISA Lille, 59800 Lille, France; urbi.pathak@student.yncrea.fr; 5 Department of Food Technology, Shyama Prasad Mukherji College for Women, University of Delhi, Delhi 110026, India; dipti@spm.du.ac.in; 6 Department of Molecular and Translational Medicine, Division of Pharmacology, University of Brescia, 25123 Brescia, Italy

**Keywords:** *Artocarpus lakoocha* Roxb., *Artocarpus heterophyllus* Lam., antioxidant properties, DMPD radical, antimicrobial properties

## Abstract

*Artocarpus heterophyllus* Lam. (*AH*) and *Artocarpus lakoocha* Roxb. (*AL*) are two endemic plants that grow on the Asian continent. To date, their applications have been aimed at using their fruit as a food source or for some of their therapeutic virtues. In this study, attention was given to the flowers of *AH* and *AL*. Initially, the cytotoxicity of the phytoextracts was assessed, and the content of minerals, phenols, and flavonoids was determined. Furthermore, some antioxidant components were identified by HPLC. Furthermore, the ability of *AH* and *AL* extracts to modulate the gene expression of some targets involved in the antioxidant response was studied. The results obtained highlighted the nutritional and antioxidant value of the *AH* and *AL* flower extracts. This study will contribute to enhancing the use of *AH* and *AL* flowers as potential supplements in human nutrition.

## 1. Introduction

The genus *Artocarpus* consists of tropical plants of the Moraceae family that are mainly cultivated in Asian countries such as India, Bhutan, Sri Lanka, Thailand, Bangladesh, and Malaysia [1,2]. *Artocarpus* includes almost 60 genera and more than 1000 species. This genus is believed to produce a high yield along with an ample number of nutritional components. Its species are used as a source of food and also in traditional medicinal practices [3].

**Figure 1:**
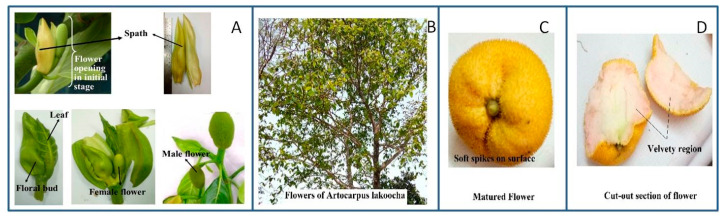
(**A**) *Artocarpus heterophyllus* Lam. flowers; (**B**) tree of *Artocarpus lakoocha* Roxb. with flowers; (**C**) upside view of *Artocarpus lakoocha* Roxb. flower; (**D**) cut-out section of *Artocarpus lakoocha* flower.

*Artocarpus heterophyllus* Lam. (*AH*), popularly known as Ceylon jack tree or jackfruit, is considered to be “poor man’s food”, as it is widely available in summer at an economical price when there is a shortage of agricultural produce in India [4,5]. In India, it is also called “Panasa”, “Atibruhatphala”, “Kantaphal”, and “Kanthal” due to its physical characteristics. The *AH* tree is also considered to be a source of flavonoids, sterols, volatile acids, carotenoids, and tannins, which vary depending on the stage of maturity [6,7,8,9,10]. Moreover, it also has a unique combination of iron, vitamin C, vitamin B complex (especially vitamin B_6_, niacin, folic acid, and riboflavin), and minerals (mainly calcium and potassium) [11,12]. The fruit of *AH* has been employed in value-added products such as fruit juice, dried chips, jam, candies, jelly, marmalades, leather, and ice cream, depending on the sensorial characteristics at different stages [11]. *AH* bears flowers after 6–8 years of planting, and flowers start to bloom from November to March. It is reported that, being a monoecious tree, the flowers are covered by two spathes of 5–10 cm in length [13] (Figure 1A). The central and peripheral regions bear male flowers, while footstalks from the main trunk bear female flowers (generally round in shape). The distinction between the flowers can be made based on surface characteristics: female flowers are generally large with a rough surface, whereas male flowers are small with a smooth surface (Figure 1A).

*Artocarpus lakoocha* Roxb. (*AL*) is another important species of *Artocarpus.* It is commonly known as “lakuchi” in India, “lokhat” in Thailand, and “tampang” in Malaysia. The tree of *AL* is mostly known for its wood and edible fruits due to its excellent nutraceutical properties [14]. In the foothills of the Himalayas in Nepal, the leaves and flowers of this tree also act as fresh fodder for lactating animals due to its excellent source of bioactive compounds [15,16,17]. Traditionally, it is also taken as medicine for people suffering from liver-related diseases [18]. The edible fruits and flowers of *AL* are consumed as vegetables, pickles, and chutney due to its astringent and acidic flavor [14]. The extract and bioactive compounds from the bark, leaves, seeds, and pericarp of *AL* fruit have been shown to possess exceptional phytochemical, nutritional, and valuable pharmacological properties [16,19,20,21]. This fruit is capable of offering numerous inhibitory factors such as antibacterial, antitubercular, antiviral, antifungal, antiplatelet, antiarthritic, tyrosinase inhibitory, cytotoxicity, and H_2_O_2_ scavenging activity, as well as exceptional activities against herpes simplex virus (HSV) and human immunodeficiency virus (HIV) [1,22,23,24,25,26,27]. The flowering pattern of *AL* is similar to *AH*, where both male and female flowers appear on the same tree. The flowers are generally irregularly round with a yellow velvety surface. The male flowers are orange-yellow, while female flowers are reddish [28] (Figure 1B–D).

In general, in both the studied species, the male flowers appear first, and the female flowers come out after and are immediately fertilized. In order not to have the interference of pollination, in this work, it was decided to use only male flowers.

Most of the studies on this subject have explored the ripe fruit of both *Artocarpus lakoocha* Roxb. and *Artocarpus heterophyllus* Lam. [1,19]. However, scant attention has been paid to the flowers of these plants. As is known, the flowers can contain many secondary metabolites with high antioxidant and antimicrobial activities [29]. Therefore, the present study was undertaken to characterize and compare the flowers of *Artocarpus lakoocha* Roxb. and *Artocarpus heterophyllus* Lam. concerning their bioactive properties. Analyses of the content of flavonoids and phenolic compounds were performed on fresh starting material. This allowed us to establish the action of the entire plant phytocomplex. Indeed, the use of fresh extract allows one to keep the biological characteristics of the plant sample unaltered at the time of sampling [30]. Various environmental variables associated with the harvest or post-harvest can induce the plant to modify, i.e., increase or decrease, some biologically active secondary metabolites such as flavonoids and phenolic compounds [30]. Other tests, such as toxicity and antioxidant activity, were determined on a dry extract obtained with different solvents (water, ethanol, and methanol). In addition, the expression of some target genes involved in antioxidant pathways and the preliminary antimicrobial action were evaluated.

## 2. Results

### 2.1. Chemical Properties of Flowers

#### 2.1.1. Mineral Composition of Flowers

The mineral content of the *AL* and *AH* flowers was analyzed by atomic absorption spectroscopy, and the most abundant minerals are presented in Table 1. Generally, the *AH* flower has a considerable amount of mineral content compared with the *AL* flower. The highest concentration of minerals in the *AL* flower was found to be copper (597.10 μg/100 g), zinc (421.06 μg/100 g), manganese (250 μg/100 g), and magnesium (13.40 mg/100 g), whereas calcium (25.35 mg/100 g), potassium (214.56 mg/100 g), and phosphorus (19.63 mg/100 g) were found to be higher in *AH*. Lower concentrations of phosphorous (11.85 mg/100 g), calcium (20.24 mg/100 g), iron (108.5 μg/100 g), and potassium (150 mg/100 g) were observed for *AL*, whereas magnesium (7.28 mg/100 g), copper (106.62 μg 100/g), manganese (185 μg/100 g), and zinc (320.22 μg/100 g) were found for the *AH* flower. These results show a significant difference between *AL* and *AH* at the level of mineral content. Indeed, both flowers contain appreciable amounts of essential minerals, which can contribute to the nutritional value of these flowers.

#### 2.1.2. Phytochemical Composition of Flowers

The total phenolic content (TPC) and total flavonoids content (TFC) of both flowers are presented in Table 1. It is evident from the results that *AH* contains a higher amount of TPC and TFC compared with the *AL* flower. In general, the *AH* flower had 883.20 μg gallic acid equivalent (GAE)/g of phenolic content, whereas only 217.80 μg GAE/g of phenolic content was found in the *AL* flower. Furthermore, the total flavonoids content was 658.52 and 168.26 μg quercetin equivalent (QE)/g in *AH* and *AL* flowers, respectively. Particularly, phenols are synthesized in the plants in response to abiotic stress and injuries by insects or mammals. In a study performed by Li et al. [31], they reported the phenolic content of 51 flowers in the range of 34.14–1.11 mg GAE/g. The results of the present study are well within the range of phenolic content reported by previous authors. Many factors influence the phenolic content, which include, mainly, the botanical source and extraction methods [32]. From the results, it could be easily inferred that *AH* flowers exhibit higher antioxidant potential than *AL* flowers.

The total carotenoids content was found to be higher in the *AH* flower (27.29 μg/g) compared with the *AL* flower (16.96 μg/g). Carotenoids are metabolized in the liver into vitamin A, which helps protect the body from damage caused by oxidative stress and inflammation [33]. The TPC and TFC content of *AL* and *AH* flowers can vary depending on the solvent used for extraction and the origin of the products [34]. Indeed, the edible flower samples from China reported total phenols in terms of gallic acid (1177.8–27,717.2 μg/g), total flavonoids in terms of quercetin and hesperidin (49.9–14,576.6 μg/g), and quercetin and luteolin (8.8–480.0 μg/g) [35].

**Figure 2:**
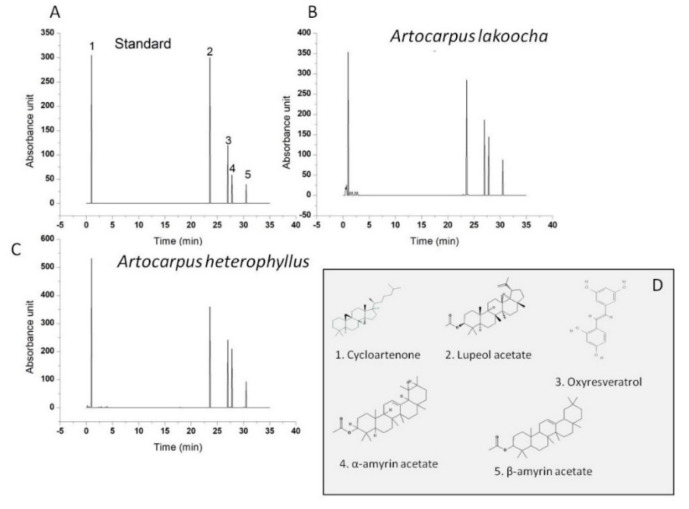
(**A**) HPLC chromatogram of standards identified in *Artocarpus lakoocha* Roxb. and *Artocarpus heterophyllus* Lam.: cycloartenone (**1**), lupeol acetate (**2**), oxyresveratrol (**3**), α-amyrin acetate (**4**), and β-amyrin acetate. (**B**) HPLC chromatogram of *Artocarpus lakoocha* flower extracts. (**C**) HPLC chromatogram of *Artocarpus heterophyllus* Lam. flower extracts. (**D**) The chemical structure of the main components.

TPC and TFC analyses were performed on fresh extracts of the flower, while HPLC analysis was performed on a dry extract. Indeed, from previously reported studies, it was observed that oxyresveratrol, α and β amyrin acetate, lupeol acetate, and cycloartenone are major compounds found in different parts of the plant of both flowers, such as the stem, heartwood, pericarp, and bark [36]. The potential benefits of these compounds have been reviewed by several authors and include anti-hyperglycemic, hypolipidemic, antiatherosclerotic, anti-inflammatory, radical scavenging, and anti-hyperlipidemic activities [37,38,39]. In order to identify these compounds, aqueous extracts of both flowers were evaluated by HPLC analysis (Figure 2).

### 2.2. Cytotoxicity Assay (3-(4,5-Dimethylthiazol-2-yl)-2,5-Diphenyltetrazolium Bromide (MTT))

**Figure 3:**
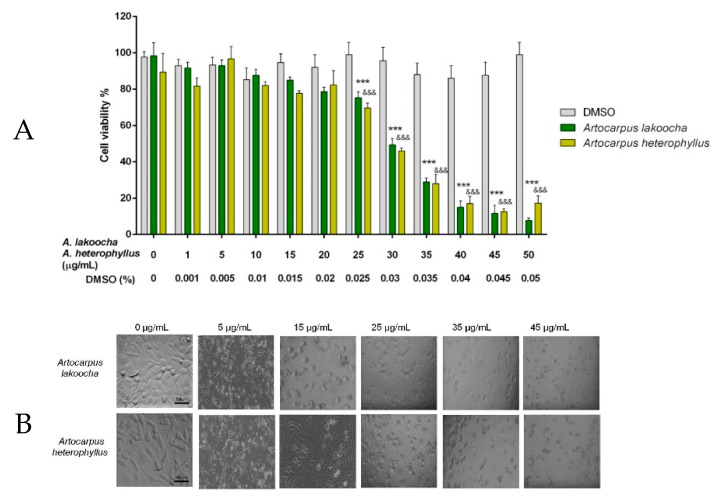
Effects of flower aqueous extracts of *Artocarpus lakoocha* Roxb. and *Artocarpus heterophyllus* Lam. on Caco-2 cell viability. (**A**) The bars indicate percent cell viability of cells treated with one of the two floral extracts with the indicated concentrations (ranging from 0 to 50 μg/mL) for 48 h and subjected to a 3-(4,5-dimethylthiazol-2-yl)-2,5-diphenyltetrazolium bromide (MTT) assay. Data are representative of three replicates and shown as mean ± standard deviation; *** *p* < 0.001 vs. *Artocarpus lakoocha* Roxb. untreated group; ^&&&^ *p* < 0.001 vs. *Artocarpus heterophyllus* Lam. untreated group. (**B**) Effects on Caco-2 cell cytotoxicity after 48 h treatment with aqueous solution extracts of *Artocarpus lakoocha* Roxb. and *Artocarpus heterophyllus* Lam. Only concentrations of 0, 5, 15, 25, 35, and 45 mg/mL are shown in the image.

Cytotoxicity is a critical aspect to measure the therapeutic effect of a plant or any synthesized new material, which should produce effects only on the targeted cells without harming the host body. Thus, the biocompatibility of these flower extracts was evaluated in terms of the % viability of Caco-2 cells. Caco-2 cells were treated with the *AL* or *AH* flower extracts at different concentrations ranging from 0 to 50 μg/mL for 48 h. The results from the 3-(4,5-dimethylthiazol-2-yl)-2,5-diphenyltetrazolium bromide (MTT) assay (Figure 3) showed that both extracts had some cytotoxic effect on Caco-2 cells in a concentration-dependent manner. The IC_50_ value of the *AL* and *AH* flower extracts was 28.43 and 29.37 μg/mL, respectively. All dosages above 35 μg/mL resulted in suffering and cell death. On the contrary, *AL* and *AH* flower extracts at concentrations below 20 μg/mL did not affect cell viability. DMSO (vehicle control) at all concentrations (0–0.05%) relevant to the treatment group showed no apparent cytotoxicity to Caco-2 cells. Since, in terms of cell viability, the floral extracts of *AL* and *AH* in ethanol, methanol, and water highlighted the same results (data not shown), only the results obtained from the extracts in aqueous solution are shown in Figure 3.

### 2.3. Antioxidant Properties

There are many factors that influence the antioxidant potential of plant extracts, including the extraction method, the composition of the extract, and the procedure used to determine that [40]. There is no precise or standard extraction method, even for estimation of antioxidant potential. Hence, it is mandatory to evaluate the antioxidant potential by taking into account more than a single analytical method. Because the mechanism of antioxidant action differs by method, in the present study, the antioxidant potential of both flowers was measured by three different methods on the basis of free radical scavenging and ferric-reducing activity, which are 2,2-diphenyl-1-picrylhydrazyl (DPPH), ferric-reducing antioxidant power (FRAP), and N, N-dimethyl-p-phenylenediamine (DMPD). Furthermore, the antioxidant action of the flower extracts was evaluated on the mRNA expression of nuclear factor (erythroid-derived 2)-like 2 (Nrf2), heme oxygenase-1 (HO-1), and NAD(P)H quinone reductase (NQO1).

#### 2.3.1. DPPH Free-Radical-Scavenging Activity

**Figure 4:**
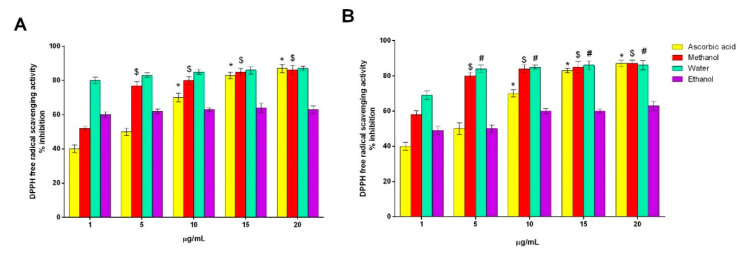
2,2-diphenyl-1-picrylhydrazyl (DPPH) free-radical-scavenging activity: (**A**) *Artocarpus heterophyllus* Lam. flower extracts, (**B**) *Artocarpus lakoocha* Roxb. flower extracts. All measurements were performed in triplicate. Data are shown as the mean ± standard deviation, and one-way ANOVA with a Newman–Keuls post-test was used for statistical significance; * *p* < 0.05 vs. 1 µg/mL ascorbic acid; ^$^ *p* < 0.05 vs. 1 µg/mL methanol extract; ^#^ *p* < 0.05 vs. 1 µg/mL water extract.

DPPH is a violet-colored dye that is scavenged by antioxidative compounds, and a color change is absorbed, which can be quantified spectroscopically at a wavelength of 515–528 nm. It is a widely used method for the quantification of free-radical-scavenging activity. However, this method is sensitive to light, oxygen, pH, and type of solvent used [5]. The DPPH scavenging activity of *AH* flower extracts and *AL* extracts in the concentration range of 1–20 µg/mL is presented in Figure 4A,B, respectively. All the assessed extracts were able to reduce the stable, purple-colored DPPH radical reaching 50% of reduction, except the ascorbic acid extract of both the samples at 1 µg/mL and the ethanolic extract of the *AL* extract at 1 µg/mL. A general trend was observed where increasing the concentration increased the scavenging activity in both the samples for all the solvents, except that for ethanolic extracts in *AH* flower extracts, which remained almost equal even after increasing the concentration. This antioxidative property can be attributed to several bioactive compounds present in the plants, such as carotenoids as well as phenolic and flavonoid compounds. From the above results, the aqueous and methanolic extracts at higher concentrations of both the samples proved their efficiency as an antioxidant. Similar results were observed in the aqueous and methanolic extracts of the fruits of *AH* and *AL* [5].

#### 2.3.2. FRAP Assay

**Figure 5:**
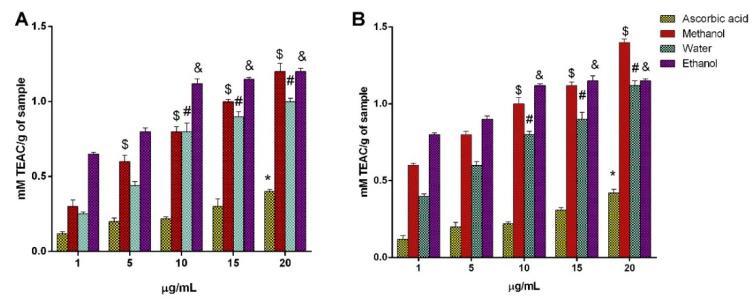
Ferric-reducing antioxidant power (FRAP) assay: (**A**) *Artocarpus heterophyllus* Lam. flower extracts, (**B**) *Artocarpus lakoocha* Roxb. flower extracts. All measurements were performed in triplicate. Data are shown as the mean ± standard deviation, and one-way ANOVA with a Newman–Keuls post-test was used for statistical significance; * *p* < 0.05 vs. 1 µg/mL ascorbic acid; ^$^ *p* < 0.05 vs. 1 µg/mL methanol extract; ^#^ *p* < 0.05 vs. 1 µg/mL water extract; ^&^ *p* < 0.05 vs. 1 µg/mL ethanol extract.

The FRAP assay, which is one of the simplest, rapidest, and most inexpensive tests, is very useful for routine analysis and is used to directly test the total antioxidant power of a sample [5]. It is based on the ferrous-to-ferric reduction potential and is considered to be an indicator of electron-donating activity [41]. The antioxidant activities of the *AH* and *AL* flower extracts using the FRAP assay in the concentration range of 1–20 mg/mL are shown in Figure 5A,B, respectively. The ferric-reducing power of both extracts increased as the concentration increased. The *AH* flower extract showed the highest ability to reduce Fe^3+^ to Fe^2+^ at a value of 1.4 mM Trolox equivalent (TEAC)/g for the methanolic extract at 20 µg/mL, and ethanolic extract of *AL* at 20 µg mL^−1^ reduced 1.4 mM TEAC/g. This revealed that a methanol soluble factor was most likely responsible for reducing the potential of the extract in the case of *AH* flower extracts and an ethanol soluble factor in that of *AL*. In general, *AH* flower extracts showed the maximum FRAP activity compared with *AL* flower extracts and reached 1.4 mM TEAC activity at the concentration of 20 µg/mL, while the other showed 1.2 mM TEAC activity. However, there was not much difference in both flowers in terms of activity at maximum concentration. This can be significantly correlated to the TPC and TFC content of both flowers. The antioxidant potential of plant products is significantly influenced by the amount of phenolic compounds and the carotenoids content. Indeed, dark-colored flowers exhibit stronger antioxidant potential than light-colored flowers due to the variation in the level of flavonoids [42]. From the present results, the carotenoids’ level significantly contributed to the antioxidant potential along with the phenolic and flavonoids content.

#### 2.3.3. DMPD Radical Cation Decolorization Assay

**Figure 6:**
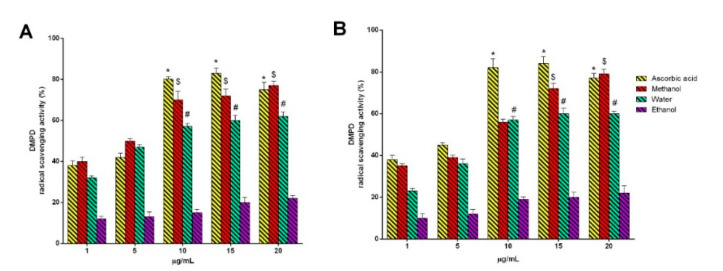
DMPD (N, N-dimethyl-p-phenylenediamine) radical cation decolorization assay: (**A**) *Artocarpus heterophyllus* Lam. flower extracts, (**B**) *Artocarpus lakoocha* Roxb. flower extracts. All measurements were performed in triplicate. Data are shown as the mean ± standard deviation, and one-way ANOVA with a Newman–Keuls post-test was used for statistical significance; * *p* < 0.05 vs. 1 µg/mL ascorbic acid; ^$^ *p* < 0.05 vs. 1 µg/mL methanol extract; ^#^ *p* < 0.05 vs. 1 µg/mL water extract.

Jagtap et al. [5] stated that the DMPD assay has some advantages due to the high stability of the endpoint, the quick reaction time, and it being cost effective and less cumbersome. In the presence of an oxidant solution (ferric chloride) at an acidic pH, DMPD is converted to a stable and colored DMPD radical cation (DMPD**^·+^**, absorption maxima 505 nm). The data in Figure 6A,B show the antioxidant activity (% RSA) of the *AH* and *AL* flower extracts in a concentration range of 1–20 µg/mL. The positive control, consisting of ascorbic acid, confirmed its antioxidant activity. Indeed, the results indicate that the antioxidative nature is dose dependent and is highest for the ascorbic acid extract in both the samples at 20 µg/mL. These data confirm the documented antioxidant action of ascorbic acid [43,44,45,46]. Ascorbic acid works as an antioxidant with many substrates such as reactive oxygen species, and its dose dependent activity has been demonstrated on many products of plant origin [43,45,47].

From the results, it was revealed that DMPD activity was significantly affected by the method of extract, solvent type, and time of extraction. A similar observation was reported by Tai et al. [48].

#### 2.3.4. HO-1, Nrf2, and NQO1 Gene Expression

**Figure 7:**
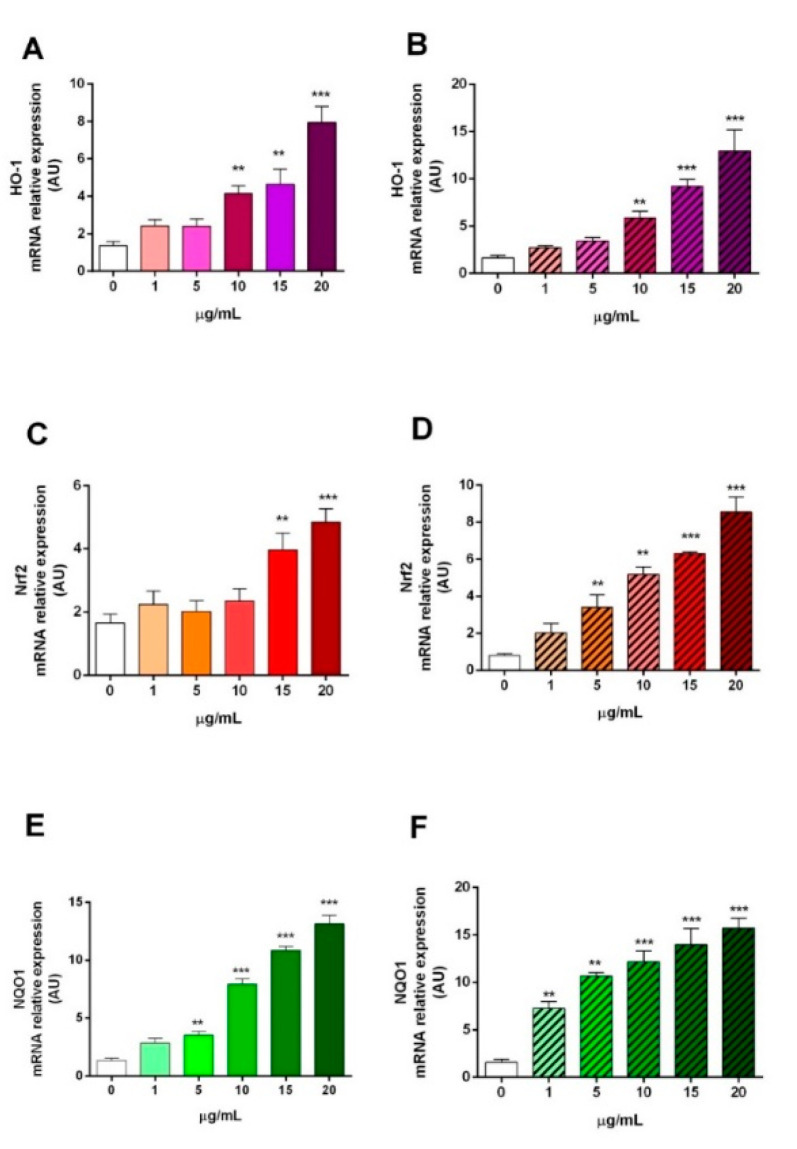
mRNA expression in Caco-2 cells of heme oxygenase-1 (HO-1) (**A** and **B**), nuclear factor (erythroid-derived 2)-like 2 (Nrf2) (**C** and **D**), and NAD(P)H quinone reductase (NQO1) (**E** and **F**) after treatment with flower extracts of *Artocarpus heterophyllus* Lam. (left) and *Artocarpus lakoocha* Roxb. (right), 0–20 µg/mL. Data are expressed as fold changes of the target gene (Nrf2 in **A**, HO-1 in **B**, and NQO1 in **C**) normalized to the internal standard control gene (β-actin). All measurements were performed in triplicate. Data are shown as the mean ± standard deviation, and one-way ANOVA with a Newman–Keuls post-test was used for statistical significance; ** *p* < 0.005; *** *p* < 0.0001 vs. 0 µg/mL.

Since in the enzyme systems described above, the two extracts showed some antioxidant activity, the aqueous extracts of *AH* and *AL* were used to evaluate the mRNA expression of Nrf2, HO-1 and NQO1 (Figure 7). In general, in both extracts, the dosages 15 and 20 µg/mL induce the mRNA expression of the three target genes compared to the control. In addition, both the extracts of *AH* and *AL* flowers increase the expression of HO-1 compared to the control starting from 10 µg/mL. Furthermore, it is interesting to note how the *AL* extracts increase the expression of Nrf2 compared to the control already at 5 µg/mL. Finally, only the *AL* extract increases the expression of NQO1 compared to the control at all dosages (1–20 µg/mL). Many authors have abundantly described the involvement of Nrf2, HO-1 and NQO1 in the processes that regulate the antioxidant response [49,50,51]. Nrf2 is a transcription factor that regulates the gene expression of many antioxidant cytoprotective and detoxifying enzymes through a promoter sequence known as the antioxidant response element (ARE) [52]. ARE is a promoter element found in many cytoprotective genes, and, therefore, Nrf2 plays a fundamental role in the cellular defense system against ARE-dependent environmental stresses as free radicals and/or reactive oxygen species (ROS) [52]. HO-1 and NQO1 are enzymes regulated by Nrf2/ARE signaling [53]. HO-1 has important antioxidant, anti-inflammatory, anti-apoptotic effects, and also appears to have an antiatherogenic action [54]. Indeed, HO-1 has been shown to be a critical gene in cellular protection and response against pro-oxidative stimuli such as ROS oxidized phospholipids [54]. At the same time, one of the major quinone reductases in mammals is NQO1. It plays multiple roles in cellular homeostasis to oxidative stress [55]. Established roles of NQO1 include the ability to enzymatically protect cellular quinones from free radical electron attacks in redox cycles. In particular, NQO1 would function as a component of a plasma membrane redox system generating antioxidant forms of ubiquinone, vitamin E, and superoxide reductase [55]. In Figure 7, the up-regulation of Nrf2, HO-1 and NQO1 mRNA confirm a direct action of the extracts of both flowers, at dosages higher than 15 μg/mL, towards the molecular signaling of cellular defense against oxidative stress also observed in other cell models and other plant extracts [49,53].

### 2.4. Antimicrobial Properties

*AH* and *AL* flower extracts were evaluated against six food pathogenic bacterial strains. Table 2 presents the diameter of zone inhibition, where it was observed that both flower extracts exhibited strong antibacterial activities against all bacterial strains at the concentration of 200 μg/mL of flower extract. All the pathogenic strains were found to susceptible to *AL* and *AH* flower extracts with various zones of inhibition. However, both extracts showed strong antibacterial activity against *Salmonella typhimurium* at the concentration of 200 μg/mL, with a zone inhibition of 24 mm. The antibacterial action of the genus *Artocarpus* has also been observed in strains of periodontal bacteria that form biofilms as reported by Teanpaisan et al. [56]. Despite the antibacterial potential observed in these data, further in vivo studies are needed to confirm these results. In the case of *Bacillus cereus*, *AH* flower extracts were not found to be effective at both concentrations. The positive control (streptomycin) significantly affected the microbial growth.

## 3. Materials and Methods

### 3.1. Flowers and Chemicals

Matured male flowers of *Artocarpus lakoocha* Roxb. and *Artocarpus heterophyllus* Lam. were collected from the horticulture department of Tezpur University, Assam, India. All the reagents used for analysis were of analytical grade and procured from Merck-Sigma, India and Hymenia, Mumbai, India. Flowers were randomly harvested in November 2019 in the morning and divided into three equal batches, representing the replicates.

### 3.2. Preparation of Flower Extracts

In order to evaluate the biological effects of the flowers extracts of the two plants, extractions with three solvents—ethanol, methanol, and water—were performed. The flowers of *AL* and *AH* were air-dried in a shed at room temperature (26 °C) for 3 weeks, after which they were ground into a uniform powder. Three solvents (methanol, absolute ethanol, or water) were separately added to the powder in a ratio of 1:10 (powder:solvent). The maceration of the extracts was carried out at room temperature for 72 h in an incubator shaker. The solution was filtered using Whatman filter paper (0.42 μm) and stored in glass vials at −5 °C.

### 3.3. Mineral Composition

The mineral content was evaluated on the fresh extracts of the flowers of both plants. An atomic absorption spectrophotometric method was employed to evaluate the mineral composition in the fresh flower samples, where 0.5 g of powdered sample (tray dried at 40 °C for 4 h) was wet-digested using nitric acid and perchloric acid at 325 °C in an autodigester (KES 12L-VA, Pelican Equipment, Chennai, India). The digested sample was diluted and aspirated into the spectrophotometer. The amount of minerals present in the flower sample was estimated by atomic absorption spectrophotometry (Model-Ice 3500, Thermo Fischer Scientific, Mumbai, India). Flame photometry and the spectrophotometric method were used to measure the potassium and phosphorus content of the flower, respectively. A calibration curve for each mineral was prepared using the standard mineral procured from Merck-Sigma, Bangalore, India.

### 3.4. Spectrophotometric Analysis of Phytochemical Properties

The content of phenols, flavonoids, and carotenoids was determined on the fresh extract of the flowers of both plants. Total carotenoids in the flowers were estimated as per the method of Lee [17], where fresh sample (5 g) was mixed with 50 mL of a mixture of n-hexane/acetone/ethanol (*v/v*; 50:25:25) and placed in an incubator shaker (Excella E24, Eppendorf, Mumbai, India) at 25 °C for 10 min at 200 rpm. The mixture was centrifuged (Eppendorf 5430R, Hamburg, Germany) to separate the sediment and supernatant at 6500 rpm at 4 °C for 10 min. Then, the supernatant was carefully collected and made up to 50 mL with the solvent used for extraction. The absorbance of colored solution was measured at 450 nm using a precalibrated UV–Vis spectrophotometer (Cary 60, Agilent UNICO Products and Instruments Inc., Shanghai, China). The results were expressed in terms of the β-carotene equivalent.

The Folin–Ciocalteu assay was used for the determination of total phenolics present in the flower extracts and expressed in terms of gallic acid equivalent (GAE). The total flavonoids content of the flower extracts was measured and expressed in terms of quercetin equivalent (QE).

### 3.5. Cytotoxicity Study

The effects of the three plant extracts on cell viability were evaluated on human epithelial colorectal adenocarcinoma cells (Caco-2 cells). Caco-2 cells are a valid system for evaluating the biological effects of plant extracts for human use [57,58,59]. Caco-2 cells were obtained from the ATCC cell bank (Rockville, MD, USA) and were propagated in DMEM (Gibco BRL, Life Technologies, Mumbai, India) supplemented with 10% fetal calf serum, 1% sodium pyruvate, 1% L-glutamine solution, and 1% streptomycin/penicillin in a humidified atmosphere of 5% CO_2_ at 37 °C.

The effect of *Artocarpus lakoocha* Roxb. and *Artocarpus heterophyllus* Lam. flower extracts on cell viability was assayed by 3-(4,5-dimethylthiazol-2-yl)-2,5-diphenyltetrazolium bromide (MTT) to obtain the range of toxic and nontoxic concentrations. Caco-2 cells were seeded in 96-well plates at a density of 5 × 10^4^ cells per well for 24 h in complete medium. Cells were then treated with flower extracts at various concentrations (1–50 μg/mL) or with vehicle (DMSO at 0.001–0.05%) for 48 h; then, the cells were exposed to the MTT reagent (0.4 mg/mL in PBS) for 30 min at 37 °C, 5% CO_2_. The absorbance at 570 nm was measured using the microplate reader MPR A4i (Tosoh, Tokyo, Japan).

### 3.6. Antioxidant Potential

Based on the results obtained with the MTT test, the less toxic dosages (1–20 μg/mL) were used to evaluate the antioxidant power of the different extracts.

#### 3.6.1. DPPH Free-Radical-Scavenging Activity

The total antioxidant properties of both flowers were estimated in terms of DPPH free radical scavenging following the method of Blois [20] with certain modifications. Different concentrations (1–15 µg/mL) of extracts were prepared in different solvents, such as acetone, aqueous, and methanol, and the extracts (0.5 mL) were mixed with 3 mL of DPPH solution in methanol (0.1 mM). The prepared mixture was allowed to react in the dark at 37 °C for 30 min, and absorbance was measured at 517 nm. Ascorbic acid served as a positive control.

(1) $$\begin{array}{c} DPPH\;free\;radical\;scavenging\;activity\;\left(\%\;inhibition\right)\\ =\frac{A_{c}-A_{s}}{A_{c}}\times 100 \end{array}$$

where *A_c_* = absorbance of control and *A_s_* = absorbance of sample.

#### 3.6.2. Ferric-Reducing Antioxidant Power (FRAP)

Flower extracts of various concentrations (1–20 µg/mL) were prepared in different solvents for the estimation of FRAP [21]. The flower extract was mixed with FRAP reagent (2.7 mL) and made up to 3 mL with distilled water. The mixture was allowed to react in the dark for 30 min at 37 °C. The absorbance of the colored mixture was recorded at 593 nm, and the results are expressed in terms of mM Trolox equivalent (TEAC)/g of the sample.

#### 3.6.3. DMPD (N, N-Dimethyl-p-Phenylenediamine) Radical Cation Decolorization Assay

The DMPD assay was performed for the flower extracts (1–20 µg/mL), where the first DMPD solution (0.1 M) was prepared in distilled water. Then, the prepared solution (1 mL) was mixed with acetate buffer (100 mL) of 0.1 M concentration (pH 5 ± 0.2), followed by addition of ferric chloride solution (0.2 mL, 0.05 M), which resulted in the formation of purple cation radical (DMPD), and absorbance was measured at 505 nm. In a test tube, 1 μL of DMPD solution was mixed with 50 μL of extract, which was mixed uniformly at 25 °C for 10 min. The absorbance of the solution was measured at 505 nm, and the antioxidative potential was measured in a manner similar to the DPPH assay.

#### 3.6.4. Expression of Antioxidant Genes

In order to evaluate the antioxidant effects of the *AH* and *AL* flower extracts in vitro, Caco-2 cells were treated for 48 h with extract in aqueous solution (1–20 µg/mL). The dose with the maximum effect observed in the antioxidant reactions described above was chosen. Total RNA was then extracted from 5 × 10^6^ cells following a standard protocol [51,60,61,62,63]. RNA was quantified spectrophotometrically and 2 µg was back-transcribed using the SuperScript^®^ VILO ™ cDNA synthesis kit (ThermoFisher, Mumbai, India). An aliquot (3.5 µL) of cDNA was then amplified by real-time PCR.

TaqMan^®^ MGB (ThermoFisher) probes were used to evaluate the mRNA expression levels of genes involved in cellular oxidative processes. The probes were heme oxygenase-1 (HO-1) (Hs01110250_m1), nuclear factor (erythroid-derived 2)-like 2 (Nrf2) (Hs00975961_g1), and NAD(P)H quinone reductase (NQO1) (Hs01045994_m1). The beta-actin gene (Hs01060665_g1) was used for housekeeping. All samples were amplified in triplicate in 96-well optical plates (ThermoFisher) by the ABI Prism 7000 Sequence Detection instrument (Applied Biosystems) following the instrument protocol. Quantification was performed using the comparative method C (T), also called method 2 (−ΔΔ*C*T).

### 3.7. Antimicrobial Activity

The antibacterial activities of the flowers were measured against some food pathogenic strains (*Staphylococcus aureus*, *Listeria monocytogenes*, *Salmonella typhimurium*, *Bacillus cereus*, *Escherichia coli* (MTCC 1568), and *Bacillus subtilis* (ATCC 6633)). All bacteria were grown on blood agar plates. They were carried out in a plate assay as per the procedure outlined by Kiran et al. [64]. The swabbed plates were punched to create wells of 9 mm diameter, and the prepared flower extract (250 μL of different concentrations) was loaded in the well. Streptomycin was used as a positive control against food pathogens. The loaded plates were incubated for 24 h at 37 °C, and the zone of growth inhibition was used as the function of antimicrobial activity. The bacterial growth inhibition zone is expressed in millimeters.

### 3.8. HPLC Conditions

Some active components of the extract in aqueous solution were identified by HPLC. Oxyresveratrol, α and β-amyrin acetate, cycloartenone, and lupeol acetate were identified following procedures previously reported [65]. In particular, the main components of the *AL* and *AH* fresh flower extracts were separated by a Zorbax 300SB 4.6 × 150 mm C18 column, 5 μm, (Agilent, Palo Alto, CA, USA) with the Agilent 1200 system. The flower extracts were diluted with methanol at a ratio of 1:2, followed by filtration using a 0.45 μm PTFE membrane, and the filtered solution (10 μL) was injected. The mobile phase consisted of acetonitrile (A):acetic acid 0.1% (B) (*v/v*), using a gradient elution of 18–25% A at 0–10 min and 25–40% A at 10–25 min, and was pumped at a flow rate of 1.0 mL/min. This was followed by a 10 min equilibration period with initial conditions prior to injection of the next sample. The simultaneous detection of the active constituents was set at a wavelength of 200–400 nm. The standards were prepared following the same procedures for the samples.

### 3.9. Statistical Analysis

All experiments were performed in triplicate, all data are presented as the mean ± standard deviation, and a value of *p* ≤ 0.05 is considered statistically significant. The data were analyzed by two-way repeated measure ANOVA followed by a Holm–Sidak’s multiple test for the comparison of individual means. Gene expression data were analyzed by ordinary one-way ANOVA followed by a Holm–Sidak’s test. All the statistical analyses were performed using GraphPad Prism version 6.01 (GraphPad Software, San Diego, CA, USA).

## 4. Conclusions

*AL* and *AH* are plants with multiple uses in Asia. In particular, fruits are the most consumed and studied products to date. Few works have been focused on the characterization of the biological activity of phyto-extracts derived from *AH* and *AL* flowers. Here, the data collected highlight how both flowers are rich in mineral salts and antioxidant properties. In particular, iron, calcium and potassium predominate in AH, and copper, zinc, manganese and magnesium in *AL*. Given the difficulties in assimilating iron and calcium at the same time in the diet, these differences further enhance the plant extracts of *AL*. On the other hand, *AH* phytoextracts show a greater antioxidant component in the fresh extracts. These differences in content are not observed in the antioxidant enzymatic activity. Indeed, the phytoextracts of *AH* and *AL* flowers showed a strong antioxidant activity with the same order of magnitude. Furthermore, both phytoextracts increased the expression of both Nrf2 (promoter gene) and HO-1 and NQO1 (Nrf2-regulated genes). Therefore, the extracts of *AH* and *AL* flowers would actively modulate the genes involved in the expression of the redox enzymatic pathways. Finally, the antibacterial aspects of *AH* and *AL* phytoextracts cannot be excluded. These qualities need to be explored in further studies, but they indicate important potential in the microbiological field.

In conclusion, this study allowed us to evaluate the nutritional, mineral, and antioxidant potential of *AH* and *AL* flowers. Furthermore, a preliminary antimicrobial evaluation was carried out on the extracts of both flowers, which was necessary to investigate potential future applications in the microbiological field. In Asia, the fruits of *AH* and *AL* are best known for their nutritional and healthy virtues. This study showed that flowers also have important potential for human health. Furthermore, this study focused its attention only on the male flowers of both dioecious plants. Preliminary data collected on phyto-extracts of female flowers show a difference in the present chemical compounds and in biological activity.

Further studies are needed to exploit, in a sustainable way, the virtues of flowers derived from *AH* and *AL* as a supplement.

## Figures and Tables

**Table 1:** Antioxidant and mineral components of flowers.

Parameters	*Artocarpus lakoocha* Roxb.	*Artocarpus heterophyllus* Lam.	*p* Value
Total phenol content (μg GAE/g)	217.80 ± 1.25	883.20 ± 5.90	<0.0001
Total flavonoids content (μg QE/g)	168.26 ± 1.50	658.52 ± 5.60	<0.0001
Total carotenoids (μg/g)	16.96 ± 0.15	27.29 ± 0.25	<0.0001
Iron (μg/100 g)	108.35 ± 3.65	196.36 ± 8.32	<0.0001
Copper (μg/100 g)	597.10 ± 8.96	106.62 ± 5.21	<0.0001
Zinc (μg/100 g)	421.06 ± 5.12	320.22 ± 6.85	<0.0001
Manganese (μg/100 g)	250.05 ± 4.68	185.62 ± 4.55	<0.0001
Calcium (mg/100 g)	20.24 ± 2.13	25.35 ± 0.81	0.0432
Magnesium (mg/100 g)	13.40 ± 0.52	7.28 ± 0.35	0.0074
Potassium (mg/100 g)	150.00 ± 8.10	214.56 ± 4.68	<0.0001
Phosphorus (mg/100 g)	11.85 ± 0.06	19.63 ± 0.05	0.0002

Values are expressed as mean ± standard deviation (*n* = 15), a value of *p* ≤ 0.05 is considered statistically significant. The table shows the significant differences between AL and AH.

**Table 2:** Inhibition zones (mm) for common foodborne pathogens determined at 37 °C after 24 h of incubation.

	*Artocarpus lakoocha* Roxb.		*Artocarpus heterophyllus* Lam.		Streptomycin	
	100 μg/mL	200 μg/mL	100 μg/mL	200 μg/mL	100 μg/mL	200 μg/mL
*Staphylococcus aureus*	8	18	15	21	12	26
*Listeria monocytogenes*	11	19	12	19	13	23
*Salmonella typhimurium*	13	24	13	24	17	31
*Bacillus cereus*	7	12	-	-	10	18
*Escherichia coli MTCC 1568*	12	22	15	22	15	26
*Bacillus subtilis ATCC 6633*	6.5	12	5	10	8	15

Note: (-) = no activity observed.
